# Phase I/II trial of bendamustine, ixazomib, and dexamethasone in relapsed/refractory multiple myeloma

**DOI:** 10.1038/s41408-019-0219-3

**Published:** 2019-07-29

**Authors:** Binod Dhakal, Anita D’Souza, Mehdi Hamadani, Carlos Arce-Lara, Katrina Schroeder, Saurabh Chhabra, Nirav N. Shah, Katelyn Gauger, Taylor Keaton, Marcelo Pasquini, Parameswaran Hari

**Affiliations:** Division of Hematology/Oncology, Medical College of Wisconsin, Milwaukee, WI USA

**Keywords:** Phase I trials, Phase II trials

## Abstract

In this phase I/II trial, BID, bendamustine (70, 80, or 90 mg/m^2^), ixazomib (4 mg), and dexamethasone (40 mg), was administered to 28 patients with relapsed and/or refractory multiple myeloma (RRMM) exposed to bortezomib and lenalidomide and refractory to at least one. A 3 + 3 dose escalation based on dose-limiting toxicities (DLTs) was employed in phase I (total 15); 2/6 patients developed DLTs (neutropenia and thrombocytopenia) at dose level 3 establishing the recommended phase II dose as bendamustine 80 mg/m^2^, ixazomib 4 mg, and dexamethasone 40 mg. The median age was 67 years (range, 42–72), and 43% were females. Patients received a median of 4 (range, 4–9) prior lines of therapy, of which ~50% were double refractory. In phase II, total 19 patients were treated. With a median follow-up of 17 months, 11% achieved very good partial response, 50% achieved partial response, and 27% achieved stable disease. Median progression free (PFS) and overall (OS) survival were 5.2 months (95% CI, 1.96–8.3) and 23.2 months (95% CI 16.3–30.07). The most frequent adverse events were anemia, thrombocytopenia, leukopenia, nausea, diarrhea, and infections. Peripheral neuropathy was infrequent. BID is a well-tolerated and effective combination therapy for patients with RRMM.

## Introduction

With the introduction of several new classes of drugs, the survival outcomes of patients with multiple myeloma (MM) have improved considerably in the last decade^1,2^. Despite these effective treatments, the disease invariably relapses after a period, requiring continued intervention for disease control. Identification of new targets and development of novel agents against such targets are extremely important for the discovery of more effective treatments. Bendamustine, a bifunctional alkylator with antimetabolite activity, is an attractive option in MM due to its specific mode of activity, favorable toxicity profile, lack of cross reactivity with other agents, and its preclinical and clinical activity in patients resistant to alkylating agents^3–5^. In MM, bendamustine has clinical activity, both as a single agent^6^ and in combination with immunomodulators (ImIDs): thalidomide, lenalidomide, and pomalidomide^7–9^ or proteasome inhibitors (PI): bortezomib and carfilzomib^10,11^.

Ixazomib is an orally available peptide boronic acid that preferentially binds to the β_5_ subunit of the 20S proteasome^12^. Ixazomib has shown clinical activity both as single agent and in combination in newly diagnosed and relapsed/refractory multiple myeloma (RRMM)^13–15^. Ixazomib is approved in combination with lenalidomide and dexamethasone in treatment of MM patients with 1 prior therapy based on a phase III trial demonstrating improved progression free survival (PFS) compared to the control arm^16^. Proteasome inhibition has emerged as an important therapeutic strategy in MM; however, risk of peripheral neuropathy associated with bortezomib^17^ and cardiovascular toxicities^18^ associated with carfilzomib limit the use of these two major PI for prolonged periods of time among MM patients. Exploring the role of alternative PIs with non-overlapping toxicities like ixazomib is therefore a reasonable strategy for combination triplet regimens. In this phase I/II study, we assessed the safety and efficacy of the combination of bendamustine, ixazomib and dexamethasone (BID) in RRMM patients exposed to bortezomib and lenalidomide and refractory to at least one of the agents.

## Subjects and methods

### Study design

This open-label, single-center phase I/II study was designed to assess the safety, tolerability, and efficacy of oral ixazomib combined with bendamustine and dexamethasone when delivered together in a 28-day cycle in patients with relapsed and/or refractory multiple myeloma (RRMM) for a maximum of eight cycles. In the phase I portion three doses of bendamustine 70 mg/m^2^, 80 mg/m^2^, and 90 mg/m^2^ days 1 and 2 were tested in combination with ixazomib 4 mg and dexamethasone 40 mg (20 mg in patients ≥75 years) on days 1, 8, and 15, respectively. In the phase II portion, bendamustine was given at recommended phase II dose (RP2D) along with ixazomib and dexamethasone in the same dose as phase I. All patients were informed of the investigational nature of the study and provided informed consent per institutional and federal guidelines. This study was approved by the Institutional Review Board from the Medical College of Wisconsin and was registered at clinicaltrials.gov (# NCT02477215).

### Study objectives

The primary objective of the phase I portion of the study was to determine the RP2D of bendamustine when given in combination with ixazomib and dexamethasone. The primary objectives of the phase II portion were to estimate the overall response rates (ORR) of the three-drug combination. The secondary objectives included the estimation of the duration of response, survival (overall, OS and PFS) and clinical benefit rates (CBR).

### Drug administration

In the phase I portion of the study, a 3 + 3 design was employed, and dose escalation decisions were based on the dose-limiting toxicities (DLTs) occurring in cycle 1. DLTs were defined as any of the following events that were considered by the investigator to be related to therapy with bendamustine or ixazomib: grade 4 neutropenia or grade 3 neutropenia with fever ≥38.5 °C, grade 4 thrombocytopenia or grade 3 thrombocytopenia with clinically significant bleeding; DLTs also included any grade 3 or greater non-hematologic toxicity including grade ≥3 nausea that occurred despite maximal anti-emetic prophylaxis; diarrhea occurring despite maximal anti-diarrheal agents and delay in starting cycle 2 for >7 days because of lack of adequate recovery of hematologic and non-hematologic drug related toxicities. Anti-viral prophylaxis against herpes zoster was mandatory throughout the study period.

## Patients

### Inclusion/exclusion criteria

The study enrolled patients of 18 years old or older diagnosed with RRMM who had prior exposure to PI (bortezomib and carfilzomib) and ImiDs (thalidomide, lenalidomide, or pomalidomide). Patients also had to be refractory to either of the bortezomib and lenalidomide according to the International Myeloma Working Group (IMWG) definition of refractory disease (progressive disease on or within 60 days of stopping PI or ImIDs). Patients were required to have measurable disease defined as serum monoclonal protein (M-protein) of ≥1 g/dl of IgG or IgM, ≥0.5 g/dl of IgA or IgD, urine M protein ≥200 mg/24 h, or involved serum-free light chain of ≥10 mg/dl, Eastern Cooperative Oncology Group of 0–2 and adequate hematologic (absolute neutrophil count >1000/mm^3^, platelets ≥75,000 mm^3^), hepatic (total bilirubin ≤1.5 upper limit of normal, alanine/aspartate aminotransferase ≤3 times the upper limit of normal), and renal (creatinine clearance ≥30 ml/minute) function. Recipients of autologous or allogeneic stem cell transplant were eligible as long as there were no ongoing transplant related side effects.

Key exclusion criteria were grade >2 peripheral neuropathy; gastrointestinal disease or history of procedure that could interfere with the oral absorption of ixazomib; systemic treatment with strong CYP1A2 inhibitors or strong inhibitors/inducers within 14 days before the first dose of ixazomib; evidence of current, uncontrolled cardiovascular conditions; and ongoing/active systemic infection, active hepatitis B or C infection or known HIV positivity. Prior ixazomib was not allowed.

### Disease and toxicities assessments

Responses were assessed using the International Myeloma Working group (IMWG) criteria^19^. Refractory to either bortezomib or carfilzomib and lenalidomide or pomalidomide was defined as double refractory; refractory to bortezomib, lenalidomide, carfilzomib, and pomalidomide as quadruple refractory and to CD38 antibody was defined as penta refractory. Adverse events (AEs) were monitored throughout the study and were graded according to the Common Terminology Criteria for Adverse Events (CTCAE) v 4.03. The study investigators assessed disease responses.

### Statistical analysis

The phase I portion of the study was designed to identify doses of bendamustine with ixazomib and dexamethasone that were associated with an acceptable AE profile when delivered together in a 28-day cycle. The primary end point for the phase I was to assess the maximum tolerated dose (MTD). For the phase II portion of this trial, the primary end point was overall response rate (ORR) of the combination. The null hypothesis that the true response rates of <30% with weekly ixazomib and dexamethasone in RRMM^13^ was tested at 10% one sided significance level with 80% power. The sample size was calculated using a Simon 2-stage design. The six patients treated at the MTD in the phase I portion were also included in the phase II portion for overall sample size estimation. At stage I, 14 patients were enrolled on the study with a plan to continue enrollment if the observed response rate was at least 28.6% (4/14). At stage II, additional five patients (19 total) were to be enrolled and consider the combination “interesting” only if at least 6/19 (35.3%) patients achieved a response. Secondary end points of the phase II portion were: duration of response (defined as the first documented response to documented disease relapse, progression or death whichever occurs first), OS (defined as the time interval from the date of first study drug to death of date from any cause), PFS (defined as the time interval from the date of first study drug to relapse, progression or death from any cause), and CBR (defined as total responders and stable disease (SD) divided by the number of evaluable patients). Time-to-event measures were estimated using the Kaplan–Meier method.

## Results

A total of 28 patients were enrolled between October 2015 and January 2018; 15 in phase I (3 at 70 mg/m^2^, 6 at 80 mg/m^2^ and 3 at 90 mg/m^2^) and 13 in phase II. Median age of patients was 67 years (range, 42–72); 43% were females and 75% were White. The baseline characteristics of these patients are described in Table 1. Patients received a median 4 (range, 3–9) lines of therapy, which included bortezomib (100%), lenalidomide (100%), carfilzomib (43%), pomalidomide (21%), and alkylating agents (36%). Eighty nine percent of patients had undergone prior autologous stem cell transplant; 46% and 25% of patients were double and quadruple refractory patients, respectively. The refractory status to the last line of treatment before enrollment was: 10 (35%) refractory to lenalidomide, 5 (18%) to daratumumab-based regimen, 4 (14%) to carfilzomib-based regimen, 3 (11%) to cyclophosphamide based regimen, 2 (7%) to elotuzumab-based regimen, and 4 (14%) to others (1 pomalidomide, 1 to pomalidomide and bortezomib and 2 to multi-agent chemotherapy).

**Table 1:** Baseline characteristics

Variable	
Total number of patients	28
Age, median (range)	67 (42–72)
Sex	
Male	16 (57)
Female	12 (43)
Race	
White	21 (75)
Black	6 (21)
Asian	1 (4)
Isotype	
Light chain	5 (18)
Non-Light chain	23 (82)
ECOG performance status	
0	13 (46)
1	11 (39)
2	4 (14)
ISS staging at diagnosis	
I	11 (39)
II	7 (25)
III	5 (18)
Unknown	5 (18)
R-ISS at diagnosis	
I	7 (25)
II	9 (32)
Unknown	11(39)
Cytogenetics	
Standard risk	11 (39)
High risk	10 (35)
Unknown	7 (25)
Median lines of treatment	4 (3–9)
Prior lines of therapy	
Bortezomib	
Exposed	28 (100)
Refractory	18 (64)
Lenalidomide	
Exposed	28 (100)
Refractory	24 (86)
Carfilzomib Refractory	3 (11)
Pomalidomide Refractory	3 (11)
Elotuzumab Refractory	2 (7)
Daratumumab Refractory	5 (18)
Double Refractory	13 (46)
Quadruple/Penta Refractory	7/6 (25/21)
Prior stem cell transplant	
Yes	25 (89)
No	3 (11)

High risk: t (4;14), t (14:16), t 14:20), 1q gain, 1p deletion, del 17p; double refractory: refractory to lenalidomide or pomalidomide and bortezomib or carfilzomib; quadruple: refractory to lenalidomide, bortezomib, carfilzomib and pomalidomide; Penta refractory: refractory to CD38 mAB in addition to quadruple refractory

The median time from diagnosis to study enrollment was 66.5 months (range, 28–166). At the time of data cutoff, 11 (39%) of the patients had died and 17 (61%) were alive with a median follow-up of 17 months (range, 1–34).

### Dose-limiting toxicities

No DLTs were observed in at dose level (DL) 1. Given that none of the three patients experienced DLTs at the dose level 1, dose level was escalated to level 2 at which level 1 patient developed grade 4 thrombocytopenia (DLT). Given that one of three patients experienced DLT, an additional three patients were enrolled at this dose level 2 and no further DLT was observed. Following a review of toxicities on DL2, three patients were enrolled on DL3. Among the first three patients, one patient developed grade 4 thrombocytopenia. Per 3 + 3 design, DL3 was expanded to enroll additional three patients; and one patient developed grade 4 neutropenia and thrombocytopenia. As two of six patients developed DLTs at DL3, the recommended phase 2 dose (RP2D) was one dose level below at DL2 (bendamustine 80 mg/m^2^, ixazomib 4 mg, and dexamethasone 40 mg).

### Response

**Fig. 1:**
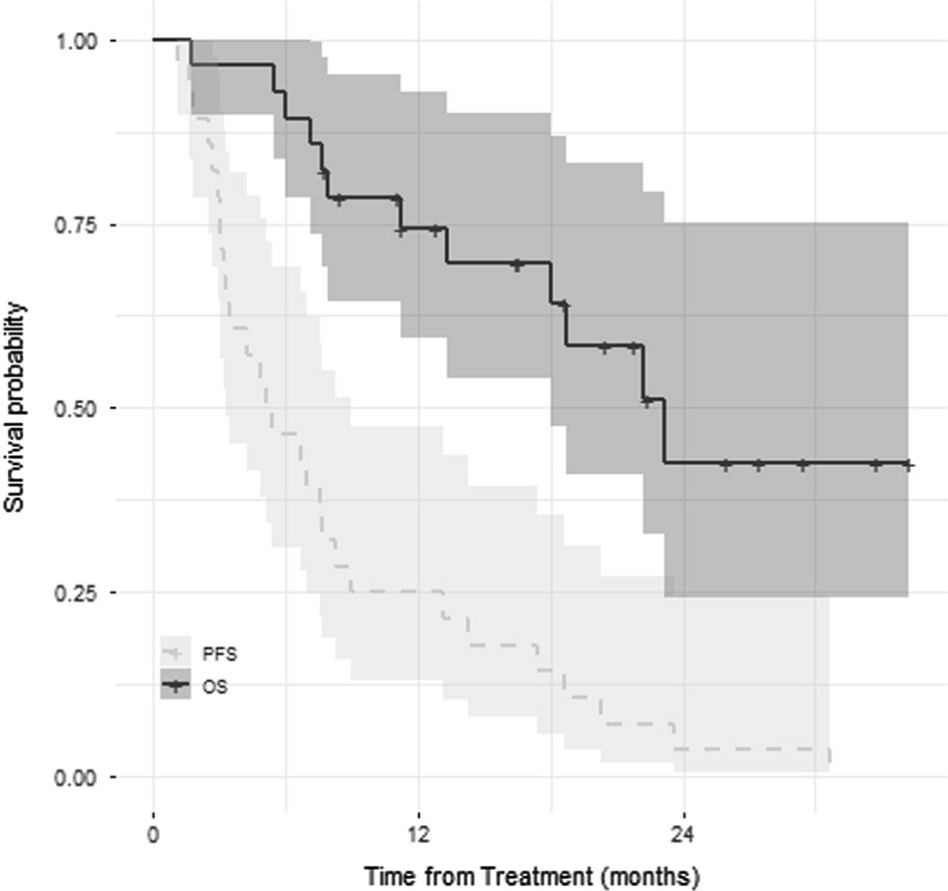
Kaplan–Meier estimates (in months) of progression free survival (PFS) and overall survival (OS) in patients treated with BID

Of the 19 patients treated at the phase 2 dosing scheme, 18 patients were evaluable for response per study definition, out of which seven completed all eight cycles. The median number of cycles completed was 4^1–8^. The most frequent reason for discontinuation before eight cycles was disease progression. The ORR was 61% with very good partial response (VGPR) in 2 (11%), partial response (PR) in 9(50%) and stable disease in (SD) 5 (27%) and progressive disease in (PD) 2 (11%) (Table 2). One patient completed less than one cycle and was not evaluable for response. For patients treated at all dose levels, the ORR was 48% with VGPR in 2 (7%), PR in 11 (41%), SD in 11 (41%), and PD in 3 (11%) (Table 2). For responders, the median duration of response was 5.2 months^2–13^. At a median follow-up of 17 months, median PFS and OS was 5.2 months (95% CI, 1.96–8.3) and 23.2 months (95% CI 16.3–30.07), respectively (Fig. 1).

**Table 2:** Response and outcomes

Variable	*N* (%) (Phase II/all patients)
SCR	0
CR	0
VGPR	2 (11)/2 (7)
PR	9 (50)/11 (41)
SD	5 (27)/11 (41)
PD	2 (11)/3 (11)
ORR	11 (61)/13 (48)
Median PFS, months (range)	5.2 (95% CI 1.9–8.3)
Median OS, months (range)	23.2 (95% CI 16.3–30.07)
Median duration of response, months	5.5 (2–9)
Median follow up, months (range)	17 (1–34)

*sCR* stringent complete response, *CR* complete response, *VGPR* very good partial response, *PR* partial response, *SD* stable disease, *PD* progressive disease, *ORR* overall response rate, *PFS* progression free survival, *OS* overall survival

### Response rates in PI exposed and refractory patients (for all dose levels)

Twelve (43%) of patients were exposed but not refractory to PI of which two (16.5%) had achieved VGPR, eight (67%) had PR, and two (16.5%) SD. Remaining 16 (57%) were refractory to PI- 3 to bortezomib only and 14 to both bortezomib and carfilzomib. For patients refractory to bortezomib only, one (33%) had PR, and two (67%) had SD. For those refractory to both bortezomib and carfilzomib, eight (57%) had SD, three (21%) PD, and one (7%) achieved PR while remaining one patient was not evaluable for response.

Twenty-four (86%) patients were refractory to ImIDs of which two (8%) had VGPR, nine (37%) had PR, 11 (46%) had SD, and two (8%) had PD. Of 13 (46%) refractory to both PI and ImIds, one (8%) had PR, nine (69%) had SD, and three (23%) had PD.

### Effect on high-risk cytogenetics

A total of 10 (35%) of patients had high-risk cytogenetics defined as presence of any of the following: t (4:14), t (14:16), t (14:20), 1q amplification, 1 p deletion, or 17p deletion (Table 1). However, of 18 evaluable phase two patients, only four (22%) had high-risk cytogenetics as defined above. The disease response for these four patients was as follows: one VGPR, one PR, and two SD. At the time of last follow-up, one patient died of disease progression and one died of progressive dementia, while two were still alive.

### Adverse events

An adverse event (AE) of any grade possibly related to treatment was reported in 100% of patients (Table 3). No treatment related deaths were observed. The most common hematological toxicities were lymphopenia (92%), thrombocytopenia (78.6%), leucopenia (61%), and anemia (57%), while the most common non-hematological toxicities included fatigue (64%), nausea (57%), diarrhea (39%), anorexia (35%), hypophosphatemia (28%), hypertension (28%), hyperglycemia (25%), hypoalbuminemia (25%), and dizziness (25%). Table 3 shows the grade 3 and 4 AEs possibly related to drug combination. Peripheral neuropathy was present in 17% of the patients and all were grades 1–2. The most common causes of death were disease progression 6(55%), pneumonia 3(27%), cardiac arrest 1 (9%), and progressive dementia 1 (9%).

**Table 3:** Hematological and non-hematological toxicities

	Dose level 1 (*n* = 3)		Dose level 2 (*n* = 19)		Dose level 3 (*n* = 6)	
	Any grade	Grade ≥3	Any grade	Grade ≥3	Any grade	Grade ≥3
Hematologic						
Neutropenia	2	1	4	3	2	1
Anemia	1	1	12	6	2	0
Thrombocytopenia	3	0	13	8	6	2
Lymphopenia	3	3	19	17	4	2
Leukopenia	2	1	12	4	3	1
Non-hematologic						
Nausea	2	0	10	0	4	0
Diarrhea	1	0	8	2	2	0
Anorexia	1	0	7	0	2	0
Increased AST/ALT	2	0	0	0	2	0
Increased creatinine	0	0	3	0	1	0
Hypophosphatemia	0	0	6	1	2	0
Hypokalemia	0	0	5	1	0	0
Hyperuricemia	2	0	4	1	0	0
Hypoalbuminemia	0	0	6	0	1	0
Peripheral sensory neuropathy	0	0	5	0	0	0
Infections	1	1	7	1	2	0
Respiratory failure	0	0	1	1	0	0
Dizziness	1	0	5	1	1	0
Hyperglycemia	0	0	7	0	0	0
Hypertension	1	0	7	3	0	0
Increased ALP	2	0	2	0	1	0

## Discussion

This prospective phase I/II trial with BID showed an impressive ORR of 61%, and clinical benefit rate of 89% in heavily treated patients with RRMM where almost half (46%) of the patients were refractory to both bortezomib and lenalidomide. The combination was well-tolerated, with manageable toxicity profile. Given the tolerability and efficacy of bendamustine alone or in combination in RRMM, the combination with ixazomib and dexamethasone required further evaluation, as it is an oral PI with low risk of neurotoxicity compared to bortezomib.

Bendamustine is an active agent in several cancers. Ex-vivo models using cell lines from mature B-cell malignancies have demonstrated the efficacy of bendamustine to trigger distinct apoptotic pathways even in cells with defective DNA repair pathway (like p53 deficient cells)^20^. In MM, this observation forms a strong rationale for bendamustine combination with drugs like bortezomib, which have shown activity in high-risk myeloma particularly 17p and t (4:14)^21,22^. Since the majority of RRMM patients are already exposed to and/or refractory to bortezomib, combining bendamustine with another PI like ixazomib makes logical sense. Additionally, ixazomib has a favorable profile including oral administration and better tolerability. When combined with bortezomib, the MTD of bendamustine ranged from 70 mg/m^2^ up to a maximum of 90 mg/m^2^ on days 1 and 2;^6,11,23^ MTD of 80 mg/m^2^ of bendamustine this study, is thus, within the range observed previously. Likewise, the dose of bendamustine varies when combined with different ImiDs as well^7,8^. Lentzsch et al. established MTD of bendamustine at 75 mg/m^2^ on days 1 and 2 in patients with median 3 prior treatment lines in combination with lenalidomide with an ORR of 50%^8^. The results of these studies point to a potential synergism of bendamustine with PI or ImiDs and formed the basis of our study.

The therapeutic efficacy of ixazomib in bortezomib RRMM patients is understudied. In an experimental in vivo model, ixazomib showed activity on cells from bortezomib resistant patients^24^. This observation has been corroborated in several clinical trials; however, the variability in response observed across studies, and the small-scale study designs preclude any definite conclusions^25,26^. Responses observed in this study were comparable to bortezomib-bendamustine-dexamethasone which resulted in ORR of 60.8% in RRMM that included patients with prior exposure, but not refractory to bortezomib^11^. The slightly lower PFS and OS observed in our study reflects the more heavily pretreated patients and a high proportion dual refractory to novel agents. Additionally, in a prespecified and post-hoc analysis of TOURMALINE-MM1 trial, the addition of ixazomib was found to overcome the poor PFS associated with high-risk cytogenetics^27^. These observations provide an impetus for further investigating the role of ixazomib in combination with alkylators and other novel agents in heavily pretreated high-risk patients.

Both bendamustine and ixazomib has been tested separately in combination with pomalidomide in lenalidomide refractory patients in phase I/II studies^9,28^. Bendamustine (at MTD 120 mg/m^2^ total dose), in combination with pomalidomide, resulted in ORR of 61% and CBR 63% in patients with median 5 prior lines of therapy^9^. The median PFS and OS of the combination was 9.6 months and 21.3 months, respectively; 18% of the patients being on planned maintenance. When combined with pomalidomide, ixazomib resulted in ORR of 53% in patients with median 2 prior lines of therapy^28^. About 2/3rd of patients in this study were refractory to bortezomib who achieved ≥PR of 29% and CBR 71% with this combination. The median PFS and OS of the combination was 8.6 months and not reached, respectively. The ORR of 61% and CBR of 91% achieved with BID regimen in this study compares favorably with the previous two studies as 64% were bortezomib refractory, 86% lenalidomide refractory, and 46% to both. Additionally, planned maintenance therapy was not used in this study and might be effective for prolonging the response duration. Furthermore, three other novel combinations reported in similar patient population are worth discussing in this context—daratumumab, pomalidomide and dexamethasone (DPd)^29^, clarithromycin, pomalidomide and dexamethasone (ClaPd)^30^ and carfilzomib, pomalidomide and dexamethasone (KPd)^31^. The ORR was 60% for DPD, ClaPD and 50% for KPD with median 4–5 prior lines of therapy. The responses observed with BID after 4 prior lines is comparable, and future study combining this regimen with CD38 monoclonal antibody is being considered.

The toxicity profile of this regimen has been similar to that seen with previously reported bendamustine or ixazomib combinations^4,14^. No grade 3 or higher peripheral neuropathy was seen in this cohort, compared to 7% grade 3 and higher seen with the bortezomib combination^11^. As seen in other ixazomib studies, we did observe gastrointestinal toxicity, particularly nausea, but this was managed with supportive care measures. Hematological toxicity remains the most common category of AEs and was similar with the ixazomib and pomalidomide^28^. Compared to bendamustine and pomalidomide combination, we observed lower rates of grade 3 and higher infections despite the higher total dose of bendamustine (160 mg/m^2^ vs. 120 mg/m^2^)^9^.

In conclusion, the combination of bendamustine-ixazomib and dexamethasone is a well-tolerated and effective combination that can be used in heavily pretreated RRMM patients. These findings justify further study of this combination in RRMM patients especially with continued ixazomib maintenance in responders^32^. Our study could also form a basis for future combination studies with ultra-novel agents like monoclonal antibodies given the lower acquisition costs associated with bendamustine.
